# Perspectives on consciousness in patients with disorders of consciousness from brain injury: group concept mapping study across clinic, research, and families

**DOI:** 10.1186/s12913-023-09438-z

**Published:** 2023-05-10

**Authors:** Niklas Blond, Lise Marie Andersen, Eva Elisabeth Wæhrens, Mette Terp Høybye

**Affiliations:** 1 Interacting Minds Centre, Department of Clinical Medicine, Aarhus University, Jens Chr. Skous Vej 4, Aarhus C, DK- 8000 Denmark; 2 Center for Elective Surgery, Silkeborg Regional Hospital, Silkeborg, Denmark; 3 The Parker Institute, Copenhagen University Hospital Bispebjerg and Frederiksberg, Copenhagen, Denmark; 4 Occupational Science, Department of Public Health, User Perspectives and Community-based Interventions, University of Southern Denmark, Odense, Denmark

**Keywords:** Brain injury, Disorders of consciousness, Group Concept Mapping, Stakeholder involvement

## Abstract

**Background:**

An effective healthcare system depends on clinic, research, and patient/relatives interactions. Such interactions may at their core be challenged by misalignments of concepts and the practices that constitute them. The concept of consciousness and what is experienced and understood as signs of consciousness in patients with severe acquired brain injury is one of these potential areas of misalignment. Different perspectives and experiences of consciousness are challenging the delivery of care and the high-stake decision-making process on the potential withdrawal of treatment. The enhanced uncertainties call for reflections on how key stakeholders perceive and identify consciousness in current clinical encounters and practice.

**Methods:**

The study empirically explores the actual experiences and conceptions of consciousness concerning patients with disorders of consciousness (DoC) from the perspectives of researchers, health professionals, and relatives of patients, to understand the challenges of the diversity of understandings of consciousness. Engaging the stakeholders by employing Group Concept Mapping methodology, the study developed a situated conceptual map, which reflects nuances and the importance of perspectives on and signs of consciousness.

**Results:**

Twenty-seven participants contributed to the generation of ideas, 14 took part in the structuring of statements and 10 took part in the validation meeting to interpret the cluster rating map. A total of 85 unique statements were identified and organized into six clusters: (1) Presence, (2) Intentional Activity, (3) Experience of self, (4) Participation in Social Interaction, (5) (Repeated) Response, and (6) Unspecific Reaction. The conceptual mapping demonstrates an extensive overlap in perspectives on consciousness among participants, prioritizing signs that are observable at the bedside.

**Conclusions:**

The study provides a first step toward a future framework for the difficult process of decision-making concerning a segment of patients with DoC. The study highlights the importance of repeatable signs of consciousness observed at the bedside and the patient’s ability to participate in social interactions, while also considering the importance of non-clinically observable signs of consciousness.

**Supplementary Information:**

The online version contains supplementary material available at 10.1186/s12913-023-09438-z.

## Background

This study provides a first step towards a future framework for the difficult process of decision-making concerning a segment of patients with disorders of consciousness (DoC) following severe, acquired brain injury. A group of patients who are marginally represented, as they are unable to actively participate in decisions of care.

An effective healthcare system performing high-quality treatment and care is dependent on interactions between the clinic, research, and patient/family. Such interactions may at their core be challenged by misalignments of concepts and the practices that constitute them. The concept of consciousness and what is experienced and understood as signs of consciousness is one of these potential areas of misalignment.

Taking theoretical outset in the assumption that knowledge is always situated in specific perspectives on the world [1], this study seeks to map different perspectives on and aspects of consciousness that may situate knowledge in particular ways concerning patients with DoC. Previous sociological and ongoing ethnographic research has shown that the everyday practices in clinics and research to assess and establish understandings of consciousness differ and are tied to different types of uncertainty about diagnostics and prognosis in patients with DoC [2–4]. As has been described, views on what may indicate the presence of consciousness in patients with DoC are nuanced and inconsistent [3, 5].

Current standard assessment tools, used in diagnosing patients with post-comatose DoC, are primarily behavioural and observational in scope and issued at the bedside, such as the JFK Coma Recovery Scale-Revised (CRS-R) [6, 7]. They, thus, focus on the overt signs of awareness of self and environment classically linked with the capacity for consciousness. The standard taxonomy of DoC includes categories along a single dimension of the level of consciousness such as Vegetative State (VS), Minimally Consciousness State (MCS), MCS+, and MCS–, as well as the Emerged from Minimally Consciousness State (EMCS) [8]. Patients in VS are understood to lack any capacity for consciousness, despite a state of wakefulness, sleep-wake cycles, and capacity for some reflective and spontaneous behaviours. Patients in MCS are understood as having some, though restricted, capacity for consciousness evidenced by clearly discernible behavioural evidence of minimal awareness of self and environment. MCS + is associated with high-level responses, such as command following, whereas MCS– requires so-called low-level behaviours only such as e.g. visual pursuit [8].

However, with the experimental advent of new technologies for diagnosis in patients with DoC, such as advances in assistive functional neuroimaging, the concept of consciousness is being challenged and negotiated in new ways and new uncertainties arise [2, 9–12]. A meta-analysis on studies applying active, passive, and resting state paradigms using assistive neuroimaging technologies such as functional MRI and EEG suggests that up to 15% of patients clinically diagnosed to be in a VS, show signs of consciousness, by command-following through modifying their brain activity during EEG or fMRI paradigms [7]. As these new diagnostic technologies are being developed, the taxonomy and prognostic assessment tools related to DoC are increasingly debated in the field as uncertainties arise and call for revisions are being put forward [6, 13, 14]. A particular concern in the field is the current taxonomical framework used concerning DoC [6, 11, 15]. Some argue that the category VS needs to be replaced by Unresponsive Wakefulness Syndrome (UWS) to embrace the uncertainty [11]. Others have called for a radical revision, challenging the level-based conceptualization of consciousness and introducing a multidimensional account of global states of consciousness such as VS and MCS, highlighting the complex nature of consciousness and diagnostics of DoC [6]. From a different ethical position, it has been argued that the categorical practice of distinguishing consciousness between VS and MSC has unintended and unwanted consequences for the patient and families, suggesting that aspirations to confirm consciousness by novel technology may be a diagnostic illusion [3].

Earlier work on moral dilemmas and conflicts experienced by health professionals around decision-making processes in the care of patients with DoC [16] supports that different perspectives and experiences of consciousness are challenging the delivery of care and decision-making process [17]. The enhanced uncertainties call for reflections on how key stakeholders perceive and identify consciousness in current clinical encounters and practice.

The perspectives and responses of families of patients with DoC are also to a great extent absent in public discourse and representations of neurotechnological and scientific advances into consciousness in DoC [18]. Such skewed public focus on scientific advances as a potential to give patients voice and choice (as e.g. argued in [9]) may not only ignore key limitations and ethical dilemmas in the clinical translation of neuro-technologies. They may also overlook a key question of whether the concept of consciousness carries the same meaning to different stakeholders, with different perspectives and in different settings around the patient with DoC.

The objective of this study is to empirically explore the actual experiences and conceptions of consciousness about patients with DoC from the perspectives of researchers, health professionals in neurocritical care, and relatives of patients, to understand the challenges of the diversity of understandings of consciousness. Hereby aiming to obtain insight into conceptual alignments and misalignments, which may challenge translational practices and decision-making when potentially moving towards implementing new technologies. Engaging the stakeholders by employing Group Concept Mapping (GCM) methodology [19, 20], this study developed a situated conceptual map, which reflects nuances and indicates which clusters hold statements of perspectives on and signs of consciousness that participants found important. This may be employed to further develop a tool to prevent conceptual misalignments about consciousness in patients with DoC that could ethically challenge the provision of care and the high-stake decision-making processes on potential withdrawal of treatment.

## Methods

### Study design and procedures

To address the aim of the study and ascertain broad perspectives on the concept of consciousness, the authors of this study applied Group Concept Mapping (GCM) methodology [19, 20]. GCM is a mixed methods approach combing qualitative production of data and methods from statistical analysis [18]. It is specifically designed to engage key stakeholders in all stages of research, the generation of data, analysis, and interpretation of the results. As a participatory collaborative process, it affords the integration of perspectives of diverse groups of stakeholders and combines these in a conceptual overview with rigor and scientific credibility [21]. There are similarities between GCM and other mixed methods strategies such as cultural domain analysis [22, 23]. For instance, both use pile sorting and multidimensional scaling techniques. However, these are different approaches in that cultural domain analysis seeks to uncover the shared meaning assigned by participants to a particular phenomenon by examining the terms that they use. The analysis focuses on the intersection of language and cultural meaning. In GCM, the goal is to capture not only participants’ shared views but also the unique views of individual participants [24]. While GCM shares the goal of and bears multiple similarities to other methods of community-based participatory research it has the benefit of being an affordable method and the flexible online format, which we employed in this study, allows individuals to participate when convenient, including taking breaks in the process [24]. This flexibility supports the involvement of more vulnerable stakeholders, though we acknowledge that participation in the online task also demands a certain level of digital competence from participants. The structured group conceptualization of GCM also has the benefit of producing a conceptual framework, which is rendered accessible in a visual format (maps). GCM has successfully been applied in the planning and evaluation of healthcare systems [25, 26].

The GCM process includes the following phases: (1) preparation, (2) generation of ideas (brainstorming), (3) structuring statements (sorting and rating), (4) performance of GCM analysis, (5) interpretation of the map (validation), and (6) utilization (developing a conceptual model) [20]. Participants in GCM studies are involved in several steps of the research process, including the generation of ideas, structuring statements, and interpretation of the maps. The GCM process may involve face-to-face group sessions, online participation, or both [19]. A pooled analysis of previous GCM studies has indicated high-reliability estimates for sorting and rating processes, as well as high representational validity [25].

In this study, the process of generation of ideas and structuring statements was conducted online between December 8^th,^ 2021, and March 24^th,^ 2022, using the Concept System® (CS®) Groupwisdom™ software, designed to support each step in the GCM process (Concept Systems Incorporated®, 2019). Interpretation of the map took place at a three-hour face-to-face validation session on May 23rd, 2022.

### Ethics approval and consent to participate

All participants were informed about our research interest at all phases of the GCM study. Participation was anonymous in all phases, except for phase 5. All participants provided information on their identification with one of the groups included and gave electronic consent to their answers to be used in research. Participants in phase 5 (validation workshop) provided additional, written informed consent to the study. The study is registered with Central Denmark Region, Denmark, research directory and follows the regional data management and protection guidelines. The Central Denmark Research Ethics Committee was notified of the project (case number: 1-10-72-274-21), which then waived the ethics approval for this study.

### Participants

Potential participants for this study were (1) researchers having research experience in neuroscience, neuro clinical or neurorehabilitation research or other related areas, (2) healthcare professionals with clinical experience from assessment of DoC when working in intensive care, neurocritical care or neurorehabilitation, and (3) relatives of an adult person with DoC following acquired brain injury.

Participants from all three groups were invited to take part in the study by email. Initially, invitations were sent out to researchers and health professionals, taking an outset in the professional network of researchers and health professionals of the research team and their institutions. Also, relatives of persons with DoC whom the research team had interacted with in previous studies were invited. Moreover, additional relatives were invited through brain injury groups on social media. To ensure a broad and diverse sample of participants across the three participant categories, snowball sampling was employed based on suggestions from invited participants.

As per the GCM approach, all participants were invited to be involved in generating ideas (phase 2) and structuring statements (phase 3). For the structuring of statements phase, additional invitations were sent out to participants at a research symposium on perspectives of consciousness held by the research team. For the interpretation of the map (phase 5), a validation workshop was conducted with invited participants from all three groups.

Members of the author group (MTH and LMA) were also invited to take part in phases 3 and 5 of the study along with the participants. The remaining authors responsible for employing (EEW) and assisting (NB) the GCM methodology (i.e., preparation, software, and data handling, the GCM analysis, and being chair (EEW) at the validation meeting) facilitated the workshop and did not take part as participants.

### GCM procedures: Data Generation

The process of GCM described above serves as a structure for the procedures in the study.

*Phase 1: Preparation for GCM* Before initiating the data collection, a focus prompt was developed and piloted, as an open-ended sentence for the participant to finish. The final version was: “By consciousness, I mean ….”.

*Phase 2: Generation of ideas (brainstorming)* Participants received an email with a link to online participation using the CS® Groupwisdom™ software. They were instructed to think broadly and generate as many ideas as possible in response to the focus prompt. They were reminded to keep each reply short and limited to only one statement.

The ideas generated were exported for consolidation; three authors; NB, LMA & MTH individually identified redundant statements (i.e., ideas with the same wording or meaning). Next, they met and discussed their findings to achieve consensus. The final set of statements was then re-imported into CS® Groupwisdom™ in preparation for phases three and four.

*Phase 3: Structuring the statements (Sorting and Rating)* Again, participants received an e-mail with a link to online participation in the sorting and rating tasks using the CS® Groupwisdom™ software. They were presented with the total number of statements and asked to organize all statements into piles, in any way that made sense to them. The only rules were: (A) there must be more than one pile, and (B) there must be fewer piles than the number of statements. Each participant was asked to label each pile of statements and rate the importance of each statement on a four-point ordinal scale: (1) *“Not at all important,”* (2) *“Somewhat important,”* (3) *“Important,”* and (4) “*Very important.*”

### Data analyses

*Demographic* In the CS® Groupwisdom™ software participants were asked to indicate whether their contribution to the study primarily was based on being a relative to a person with DoC, a health professional, or a researcher. These data are presented as counts and percentages.

*Phase 4: GCM analysis* Based on the sorting and ratings, multidimensional scaling and cluster analyses were performed, in which related statements were grouped into clusters. To ensure the quality of the overall sorting and rating data, single-participant data from phase three were included in the cluster analysis if more than 75% of the statements were sorted [19] and if fewer than ten statements remained unrated.

Within the multidimensional scaling analysis, a ‘stress value’ is the statistics used to indicate the degree to which a multidimensional scaling solution (i.e. the processed data) fits the original similarity matrix (i.e. the raw data) [25]. A stress value < 0.39 indicates goodness of fit and supports that results are interpretable [25]. During the cluster analyses, several cluster solutions were generated, and the cluster solution potentially representing sufficient details on the topic was applied, creating the Cluster Rating Map. Based on the labels provided by the participants, cluster labels were suggested by the CS® Groupwisdom™ software. The proximity of clusters on the map indicates how related they are; clusters closer together are more related than those further apart. The number of layers of a cluster signifies its relative importance, with higher clusters containing statements being rated as more important.

*Phase 5: Interpreting the map (Validating)* At the face-to-face validation session, participants met to interpret and validate the results. Based on the Cluster Rating Map and an overview of clusters and statements participants were instructed to in small groups (a) determine if each statement was placed in the right cluster, (b) consider the number of clusters, and (c) consider if the cluster labels illustrated the theme of the cluster. Only statements clearly misplaced were moved while statements fitting into more than one cluster remained in their designated cluster. Reflections and suggestions were discussed to obtain consensus.

*Phase 6: Utilizing (Developing a situated conceptual model)* Based on the validated Cluster Rating Map, a final conceptual model was developed.

## Results

In the following sections, the participants, the GCM data, and the cluster rating map are presented.

### Perspectives on consciousness

Health professionals, relatives, and researchers were involved in the GCM process to identify, organize, and prioritize understandings of the concept of consciousness. Twenty-seven participants contributed to the generation of ideas and 14 took part in the structuring of statements online. Finally, 10 participants took part in the validation meeting to interpret the cluster rating map. Table 1 presents the number of participants by study phase.

**Table 1:** Overview of participants’ involvement with stages of the Group Concept Mapping (GCM) process by participant category. GCM study of perspective on consciousness, Denmark 2022

	Generating ideas (N = 27)	Structuring statements (N = 14)	Interpreting map (N = 10)
Relative n (%)	2 (7.4)	2 (14.3)	1 (10)
Researcher n (%)	4 (14.8)	4 (28.6)	4 (40)
Health Professional n (%)	21 (77.8)	8 (57.1)	5 (50)

A total of 43 ideas were generated during the brainstorming. After splitting ideas with more than one meaning and removing redundant ideas, 85 unique statements were included for sorting and rating. Minor linguistic revisions were made to clarify statements. Eighteen participants initiated the sorting task. Of these six (33.3%) left between 1 and 78 statements (1, 8, 37, 50, 73, and 78) unsorted. When asked to rate the importance of the statements, 13 participants initiated the task. One participant left almost all (n = 83) of the statements unrated. Moreover, two participants left one and eight statements, respectively, unrated. Hence, based on the predefined criteria, the sorting of statements was approved for 14 participants, and the rating of statements was approved for 12 participants. Participants sorted the statements into between two and 18 groups (median = 5.5).

**Fig. 1:**
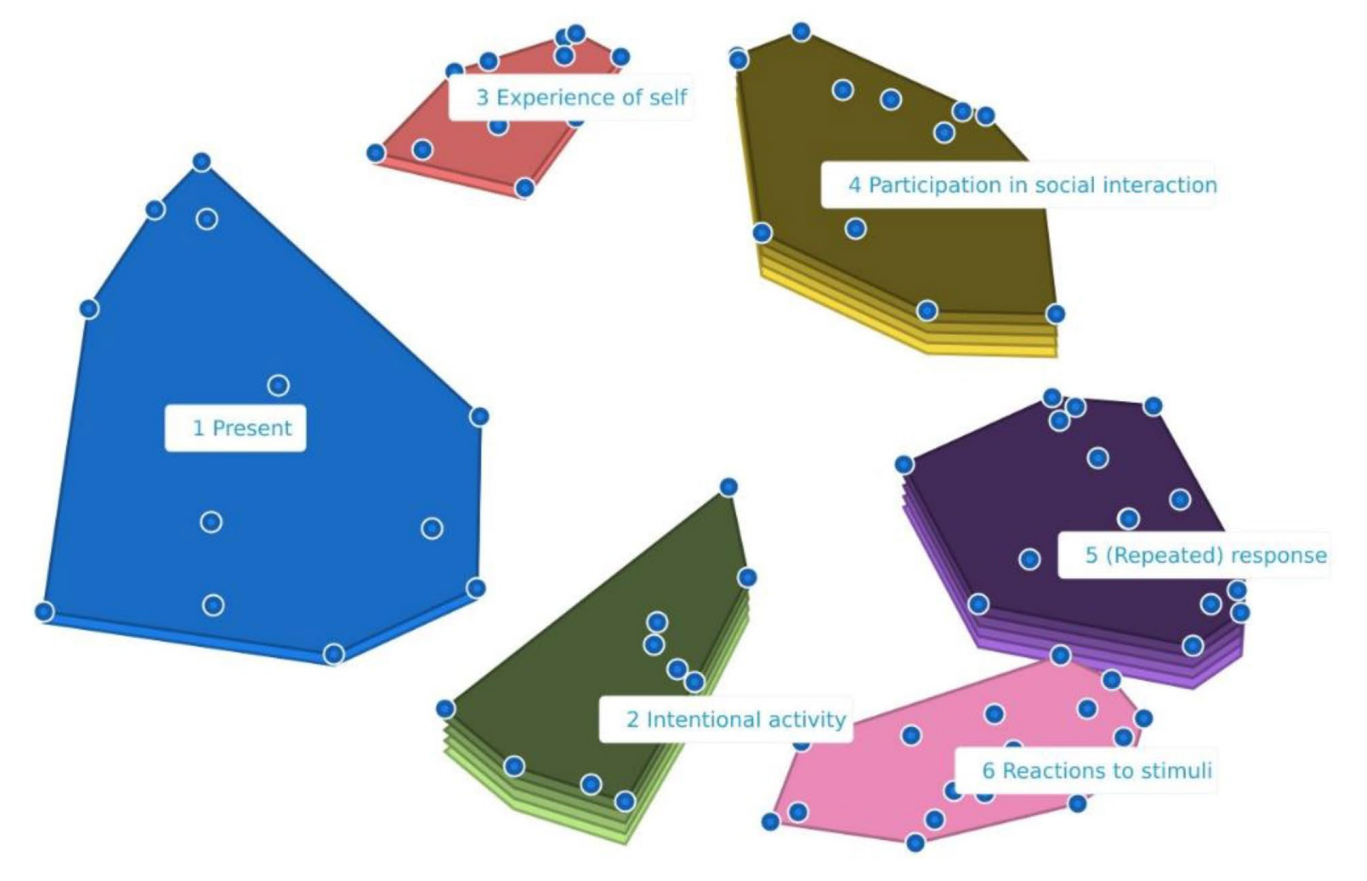
Cluster rating map with six clusters. The proximity of clusters on the map indicates how related they are. The height of a cluster indicates its relative importance, with higher clusters (i.e., the number of layers) containing statements rated as more important. The blue points represent the individual statements. GCM study of perspective on consciousness, Denmark 2022

The multidimensional scaling analysis involved ten iterations and revealed a stress value of 0.25 which indicates that the results are interpretable. In the analysis, solutions with four to eight clusters were applied. The cluster solution with six clusters, generated by the CS® Groupwisdom™ software, was chosen to be further examined at the validation meeting. The six clusters, each containing between eleven and twenty statements, are presented in a cluster rating map (Fig. 1).

### Developing consensus on signs of consciousness

For the interpretation of the map (phase 5), a validation workshop was conducted with invited participants from all three groups to interpret the cluster rating map (Fig. 1).

Discussions at the validation meeting led to consensus about the location of the majority (*n* = 75, 88.2%) of statements, and only 10 (11.8%) statements were moved between clusters (Appendix A). This process resulted in the following six key concept clusters (Table 2). Each cluster in the revised map now contained between nine and 21 statements (Table 3 and Appendix A). Furthermore, the participants suggested changes to the titles of clusters 1 (from ‘Present’ to ‘Presence’) and 6 (from ‘Reaction to stimuli’ to ‘Unspecific reaction’), based on the content of the clusters.

**Table 2:** Overview of the final six key concept clusters in the GCM analysis. GCM study of perspective on consciousness, Denmark 2022

Cluster name and number (n) of statements	Content Summary
1. Presence n = 11	Signs that reveal the presence of the patient for the beholder – signs of ‘being’ rather than ‘doing’. Such signs e.g., a wakeful state, eyes with depth, recognition, which might have great meaning for the beholder, but will be hard to use clinically.
2. Intentional Activity n = 9	Increasing clinically identifiable signs of consciousness, which can be differentiated from reflexes and appear intentional. These statements are viewed as useful for clinical assessment of consciousness, e.g., intentional eye contact, trailing motions, and selective motions distinguishable from reflexes.
3. Experience of Self n = 12	The impression of a patient’s possible inner life and which capabilities that are perceived as necessary for the patient to retain some level of consciousness. Although the cluster describes a crucial theme, e.g., being reflexive of one’s own thoughts, being able to process information, it is not directly identifiable to the observer.
4. Participation in Social Interaction n = 13	The patient’s ability to interact socially with their surroundings. Such interaction can be observed, but the meaning and relevance of interactions might differ depending on the observer. E.g., a close relative might recognise some personal traits knowing the patient’s life story, that is not readily apparent for an unwitting health professional.
5. (Repeated) Response n = 19	Cover the clinical signs of consciousness and describes the most directly observable (repeated) signs and actions that are used in assessing levels of consciousness, i.e., reproducible, non-random responses to external stimuli such as verbal requests.
6. Unspecific Reaction n = 21	Reactions to different types of stimuli, which are intentional, but not necessarily reproducible. These statements generally refer to types of tests linked to the clinical assessment of consciousness i.e., reactions to touch, sounds, pain through movement, twitches.

Generally, statements were rated as important (*n* = 47, 55.3%) or very important (*n* = 33, 38.8%) (Appendix A). This was also reflected by cluster median importance values of 3 to 4 across all clusters (Table 3), based on the final model after moving 10 statements between clusters. Still, some variation was seen. For example, in clusters 3, 4, and 5, all statements were considered either important or very important, whereas the remaining clusters also contained statements considered only somewhat or not important. This is also illustrated in the Cluster Rating Map (Fig. 1).

**Table 3:** Rating of the key concept clusters. GCM study of perspective on consciousness, Denmark 2022

Key concept clusters within the conceptual model	Importance			Number of statements
	Cluster Median^*^	Range		n (%)
1 Presence	3		2–4	11 (12.9)
2 Intentional Activity	3.5		2.5–4	9 (10.6)
3 Experience of Self	4		3–4	12 (14.1)
4 Participation in Social Interaction	4		3–4	13 (15.3)
5 (Repeated) Response	4		3–4	19 (22.4)
6 Unspecific Reaction	3		1–4	21 (24.7)

^*^The cluster median is calculated based on median values of ratings of importance on each statement within each cluster. Range statistics represent the lowest and highest median value, respectively, for ideas within a cluster

A significant part of the discussion during the validation workshop was devoted to the clinical applicability of the statements about determining levels of consciousness. As such, Cluster 1: “*Presence”* and Cluster 3: “*Experience of self”* were viewed as representing statements that concerned states of *being* rather than *doing*. Cluster 1 holds statements that were viewed as possibly significant for the beholder, but which cannot be employed in a clinical assessment. In contrast, Cluster 3 contains statements pertaining to the patient’s inner life and is thus distinct from Cluster 1, as they describe capabilities and conditions that are meaningful for the patient, but not observable by the beholder. What these clusters and their statements describe, were viewed by participants at the workshop as a prerequisite for the remaining signs of consciousness to occur.

Another main discussion revolved around whether Cluster 5: “*(Repeated) Response”* and Cluster 6: “*Unspecific Reaction”* should be merged, as they are closely related as displayed in the cluster map. Also, statements in these clusters were viewed as the most useful for clinically determining levels of consciousness. A consensus to keep the clusters separate was reached, as further discussion revealed that the statements of Cluster 5 describe consistent repeated responses, whereas the statements of Cluster 6 describe responses that may differ over time. It was agreed that Cluster 6 should change the label from *“Reaction to stimuli”* to *“Unspecific reaction”*. The discussion revealed differing levels of importance and applicability of the clusters and their statements. This also corresponded with the rating of importance made during the sorting and rating part of the GCM study suggesting that statements in Cluster 5 were rated as highly important. This indicates an agreement with the importance of repetition to relate a response to consciousness.

## Discussion

### Developing a conceptual model

**Fig. 2:**
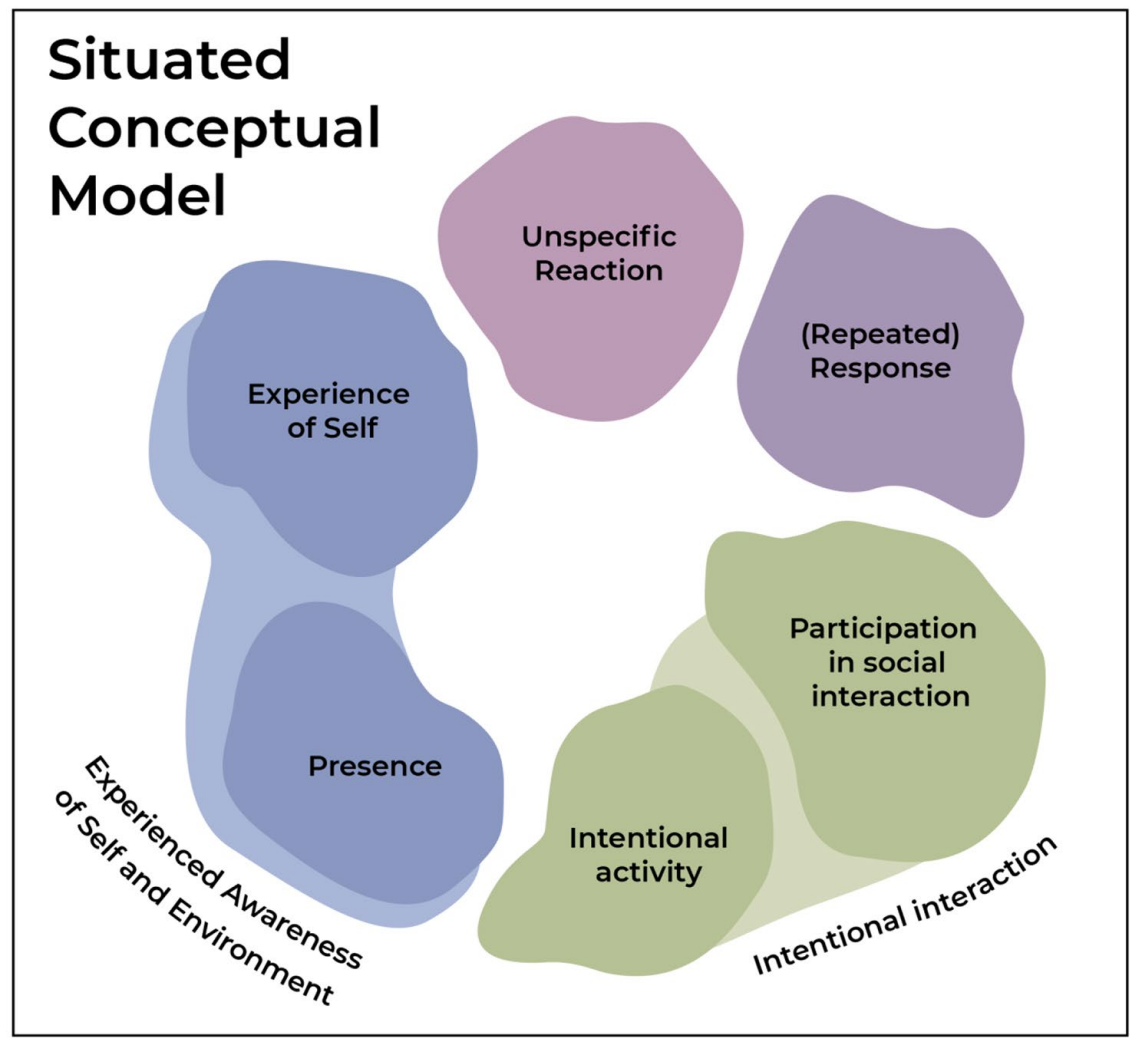
Situated conceptual model of perspectives on consciousness. GCM study of perspective on consciousness, Denmark 2022

Following and drawing on the validation discussion, a conceptual model (Fig. 2) was developed by the researchers (NB, LHA, MTH) to visualize the situated analytical points of the original cluster rating map (Fig. 1) and the clusters’ mutual connections. The situated conceptual model illustrates the six clusters, the different perspectives on consciousness that they represent, and their statements describe their mutual connections and overlaps. In line with current debates on diagnosis and the taxonomy of DoC [2, 6], we found it meaningful to create a non-hierarchical conceptual model, as it became apparent that all clusters held highly rated statements of importance. The conceptual model focuses on the conceptual overlaps between clusters as they emerged in the validation workshop discussions.

The blue theme comprising Cluster 1: *Presence* and Cluster 3: *Experience of self*, covers the least clinically measurable signs of consciousness. These clusters contain statements that may be thought of as meaningful by the onlooker but cannot be objectively reproduced (Cluster 1) or even observed by any other than the patient (Cluster 3). The theme was of importance, but not necessarily clinically relevant. The blue theme and its statements all describe basic necessity states of reaction and consciousness for the other themes in the model to be examined. The theme was thus labelled “*Experienced awareness of Self and environment*”.

The pink theme (*Unspecific reaction*) describes reactions to external stimuli e.g., observed in the form of small movements or changes in pulse. These reactions are distinguishable from reflexes but can be vague, and inconsistent and were considered by participants at the validation workshop to be on the verge of what can be perceived as signs of consciousness.

The green theme comprising Cluster 2: “*Intentional Activity”* and Cluster 4: “*Participation in Social Interaction”* describes situations that are increasingly clinically observable, while still depending on the onlooker’s relation to the patient, as we have discussed in previous research [4]. Thus, a health professional may view a social interaction with different relevance than a relative, who can mobilize knowledge from and experience of the patient’s life story, would [27]. This theme highlights the importance given to the capability of the patient being able to participate intentionally in (social) interactions, as it was considered at the validation workshop to be both foundational for the accomplishment of the remaining themes and hold great value for relatives. The theme describes reliable signs of consciousness, that are used to clinically assess levels of consciousness in patients with DoC. The theme was thus labelled “*Intentional interaction”*. About this theme, it is important to consider, as we have previously argued, that this difference may not only be a difference of perspective but also that the object of consciousness is dynamically constituted through the practices of the different actors relating to the patient [4].

Finally, the purple theme comprising Cluster 5: *(Repeated) Response* covers measurable behavioural signs of consciousness and describes a clear, repeatable response, that is distinguishable from reflexive reactions. The statements and cluster in this theme were rated at the same, high importance as clusters 3 and 4. At the validation workshop, however, participants collectively returned to the importance of the repetition that statements in this cluster represented as a key indicator of consciousness. This possibly suggests that repeated responses hold more value across disciplines and relations to the patients, but it is not supported by the cluster ratings in this study.

The cluster rating map and the situated conceptual model complement existing literature in the field by proposing a conceptual alignment of perspectives on consciousness between clinicians, researchers, and relatives [2, 6, 13, 16, 18]. The situated conceptual model sought to embed the uncertainties of the differing perspectives on consciousness across the group of participants by visualizing the conceptual thematic overlaps voiced at the validation workshop. As became evident throughout this study, both clinically asserted levels of consciousness and subjective perspectives on signs of consciousness in patients with DoC hold importance to all the involved groups.

### Implications

Developing the model, we acknowledged that different signs of consciousness hold varied levels of importance depending on the situated perspective of the onlooker. As described the themes developed during the consensus discussion at the validation workshop, which the situated conceptual model (Fig. 2) illustrates. From the consensus discussion at the workshop, it emerged that the purple theme “*(Repeated) Response*” was recognized by all participants in the workshop as containing the most important signs of consciousness. Likewise, the green theme “*Intentional interaction”* was a high-priority theme amongst all participants, sharing characteristics with the purple theme *“(Repeated Response)”* as it relates to observable interaction by the patient and thereby produces clinically measurable signs of consciousness. This possibly suggests that the repeated and intentional elements of interactions with patients with DoC hold more value across disciplines and relations to the patients, but it is a purely qualitative observation from the workshop and not supported by the GCM cluster rating.

Yet, the participants at the workshop also highlighted the importance of the blue theme “*Experienced awareness of self and environment”*, as a prerequisite for other signs of consciousness to occur. In the experience of participants, it was however not possible to determine these as clinically useful signs of consciousness. This aligns with philosophical discussions of the nature of consciousness [6, 28] as a more complex phenomenon than its overtly and clinically detectable signs. It also aligns with considerations of consciousness as multifaceted and encompassing many different aspects felt [12]. Though touching on considerations about the possible inner life of the patients with DoC, there was during the GCM process not much reflection as to whether such conscious experiences resemble that of healthy subjects, something which has been questioned in the philosophical literature [12].

Through the GCM approach, a conceptual alignment of what constitutes signs of consciousness was reached and illustrated in the situated conceptual model. The study emphasizes the importance of considering the significance of all signs of consciousness, be they clinically observable or not, as they may be perceived by the onlooker as significant, depending on their relation and history with the patient. The relational attachment to a patient may install different contexts to the interpretation of signs but may also hold different rationales about the phenomena of consciousness. The study serves as a first step to further attend to differences in perspectives and rationales across clinicians, researchers, and relatives as stakeholders in the care of patients with DoC. It illuminates the nuanced and multifaceted nature of consciousness, which may sometimes be less visible in decision-making contexts and public discussions of cases of patients with DoC. Moving forward from here, the results may be employed to further develop a tool or guideline to prevent conceptual misalignments about consciousness in future decision-making in the treatment and care of patients with DoC.

### Limitations

The participants were asked to complete the open-ended statement: “*by consciousness, I mean …”* and encouraged to think about it broadly while generating as many statements as they could. This open-ended statement was chosen to elicit answers from practitioners, researchers as well relatives and was thus open to both clinical and non-clinical perspectives on consciousness. The resulting statements focused primarily on behavioural signs of consciousness, that might be used clinically in measuring levels of consciousness. At the ensuing validation workshop, it was noted, that there was a surprising lack of non-clinical statements connected to patients’ personalised gesticulation and/or traits recognisable only to relatives e.g., a certain facial expression or bodily movement, that may express a certain mood or recognition. This could be an indicator, that most participants in the statement brainstorm were practitioners in the field, as we were less successful in recruiting relatives. It could also reflect the relative’s adoption of clinical criteria of consciousness. They often become involved in the patient’s treatment and are instructed to attend to such signs, thereby becoming accustomed to reporting them to health professionals. The context of the study and the wording of the open-ended statement could also have played a role in guiding relatives toward more clinically oriented statements.

Despite our dedicated efforts to recruit a larger number of relatives, participation from this group in the study is low. Initially, we invited relatives encountered during our research in the clinic to participate. To a large extent, however, they declined the invitation because the occurrence of the event which had induced the DoC was too recent, making participation emotionally stressful. We, therefore, changed our recruitment strategy to the engagement of online patient-relative groups on social media. While we had some success with this strategy participation was still low. Following the method of GCM we ensured that relatives were represented as a group at each stage of the method. Thus, while the greater representation of relatives would have been preferable, the perspective relatives represent does shape the situated conceptual map of consciousness and the conceptual model.

The online format of the study itself may select for participants who are confident in using digital technologies. However, this format is an integrated part of the GCM method. The fact that several phases take place online also provides for several advantages, such as flexibility concerning time and place of participation as well as allowing individuals to take breaks when necessary. A possible venue for future research is to supplement this study with a further study employing qualitative interviews to reach groups less reachable while employing the GCM method.

It was an original aim of the study to determine the relative weight of importance of diverse conceptual aspects of consciousness about the different groups participating in the study. However, we were not able to pursue this as participants did not provide their group affiliation at all phases of their participation and an absolute pattern match could not be created. Because of this and due to the overall sample size of the different groups, it was not possible to determine common perspectives from one given group. This question will be the object of future research.

While we have aimed at mapping the conceptions of consciousness as broadly as possible across perspectives and anticipate that our finding will find considerable resonance internationally, we do acknowledge that the study took place exclusively in the context of the Danish healthcare system and care structure for patients with DoC. The Danish health context and structure of care may be distinct in cases of unresponsive patients with DoC. The Danish healthcare system is guided by the principle of universal, free, and equal access to healthcare for all citizens. Due to the structure and organization of the Danish healthcare system, specialized care facilities for patients diagnosed and categorized in more persistent vegetative states do not exist, in the manner, they have been described in previous work from US care settings [29, 30] or other European settings [3, 31]. Further, there are no court actions about end-of-life decisions needed to move forward to allow for withdrawal of treatment e.g. in the United Kingdom in cases of decisions to terminate treatment [32]. Such structural and cultural differences may significantly influence conceptions and weighted importance of issues of consciousness and studies in the context of other healthcare systems are obvious avenues for further investigation.

## Conclusion

Although a significant amount of research documents the advent of new assistive functional neuroimaging technologies and the promise of the use of these in assessing levels of consciousness [6, 13], as well as a much-needed ongoing debate on the taxonomy of DoC [5, 7, 8], few studies have explored the differing perspectives on consciousness between the key stakeholders surrounding and treating patients with DoC [2, 16, 18]. In this project, GCM has been used as a method to strengthen a shared understanding across researchers working within neuroscience, neuro clinical or neuro rehabilitation research or other related areas, clinicians, and relatives attempting to provide a common conceptual map and language to prevent conceptual misalignments when encountering differing perspectives on consciousness in patients with DoC. This is highly relevant in the clinical treatment and care of these patients to ensure collaboration with relatives and agreement on decision-making processes.

The study highlights the importance of repeatable signs of consciousness observed at the bedside and the patient’s ability to participate in social interactions, while also considering the importance of non-clinically observable signs of consciousness.

## Electronic supplementary material

Below is the link to the electronic supplementary material.

Supplementary Material 1

## Data Availability

The dataset generated during and analysed in the current study is available from the corresponding author upon reasonable request.

## References

[1] Haraway D (1988). Situated Knowledges: the Science question in Feminism and the privilege of partial perspective. Feminist Stud.

[2] Andersen L, Marie HB, Boelsbjerg, Mette Terp H. Tracing uncertainties in New Prognostics of consciousness. Tidsskrift for Forskning i Sygdom og Samfund. 2020;17. 10.7146/tfss.v17i33.123589.

[3] Nettleton S, Kitzinger J, Celia Kitzinger (2014). A diagnostic illusory? The case of distinguishing between “vegetative” and “minimally conscious” states. Social Science & Medicine.

[4] Boelsbjerg H, Bess. Lise Marie Andersen, and Mette Terp Høybye. Making it count. Tracing signs of consciousness and potentiality in severe brain injury in Denmark (Currently in review in Medical Anthropology). 10.1080/01459740.2023.2300080 38206318

[5] Majerus S, Gill-Thwaites H, Andrews K, Laureys S. 2005. Behavioral evaluation of consciousness in severe brain damage. In *Progress in Brain Research*, ed. Steven Laureys, 150:397–413. The Boundaries of Consciousness: Neurobiology and Neuropathology. Elsevier. 10.1016/S0079-6123(05)50028-1. 10.1016/S0079-6123(05)50028-1 16186038

[6] Bayne T, Hohwy J, Owen AM (2017). Reforming the taxonomy in disorders of consciousness. Ann Neurol.

[7] Kondziella D, Christian K, Friberg VG, Frokjaer M, Fabricius, Møller K (2016). Preserved consciousness in vegetative and minimal conscious states: systematic review and meta-analysis. J Neurol Neurosurg Psychiatry.

[8] Royal College of Physicians (2020). Prolonged disorders of consciousness following sudden onset brain injury: National clinical guidelines.

[9] Peterson A, Mintz K, Owen AM (2022). Unlocking the Voices of patients with severe Brain Injury. Neuroethics.

[10] Lazaridis C, Syd L, Johnson M (2018). The sources of uncertainty in Disorders of consciousness. AJOB Neurosci.

[11] Laureys S, Celesia GG, Cohadon F, Lavrijsen J, José León-Carrión, Sannita WG et al. Leon Sazbon,. 2010. Unresponsive wakefulness syndrome: a new name for the vegetative state or apallic syndrome. *BMC medicine* 8: 68. 10.1186/1741-7015-8-68. 10.1186/1741-7015-8-68 PMC2987895 21040571

[12] Klein C (2017). Consciousness, intention, and command-following in the vegetative state. Br J Philos Sci.

[13] Kondziella D, Cheung MC, Dutta A (2019). Public perception of the vegetative state/unresponsive wakefulness syndrome: a crowdsourced study. PeerJ.

[14] Farisco M, Pennartz C, Annen J, Cecconi B, Kathinka Evers (2022). Indicators and criteria of consciousness: ethical implications for the care of behaviourally unresponsive patients. BMC Med Ethics.

[15] Schiff ND. Cognitive Motor Dissociation following severe brain injuries. JAMA Neurology 72 American Medical Association. 2015;1413–5. 10.1001/jamaneurol.2015.2899. 10.1001/jamaneurol.2015.2899 26502348

[16] Span-Sluyter AMFH, Lavrijsen JCM, van Leeuwen E, Koopmans RTCM. 2018. Moral dilemmas and conflicts concerning patients in a vegetative state/unresponsive wakefulness syndrome: shared or non-shared decision making? A qualitative study of the professional perspective in two moral case deliberations. *BMC medical ethics* 19. England: BioMed Central Ltd: 10–10. 10.1186/s12910-018-0247-8. 10.1186/s12910-018-0247-8 PMC5824545 29471814

[17] Rodrigue C, Riopelle RJ, Bernat JL (2013). Perspectives and experience of Healthcare Professionals on diagnosis, prognosis, and end-of-life decision making in patients with Disorders of consciousness. Neuroethics.

[18] Samuel G, Kitzinger J (2013). Reporting consciousness in coma: media framing of neuro-scientific research, hope, and the response of families with relatives in vegetative and minimally conscious states. JOMEC J.

[19] Kane M, Trochim WMK, Trochim W, Sage Publications. Concept Mapping for planning and evaluation. Applied Social Research Methods. SAGE Publications; 2007.

[20] Kane M, and Scott Rosas. Conversations about group concept mapping: applications, examples, and enhancements. SAGE publications; 2017.

[21] Trochim W (2005). Concept mapping: an introduction to structured conceptualization in health care. Int J Qual Health Care.

[22] Collins CC, Dressler WW (2008). Cultural Consensus and Cultural Diversity: a mixed methods investigation of Human Service Providers’ Models of domestic violence. J Mixed Methods Res.

[23] Israel BA, Eng E, Schulz AJ, Parker EA (2005). Methods in community-based Participatory Research for Health.

[24] Windsor LC. Using Concept Mapping in Community-Based Participatory Research: A Mixed Methods Approach. J J Mixed Methods Res. 7(3):274–93. doi: 10.1177/1558689813479175. 10.1177/1558689813479175 PMC4638322 26561484

[25] Rosas SR (2012). Quality and rigor of the concept mapping methodology: a pooled study analysis. Eval Program Plan.

[26] Ogden K, Greenfield D (2017). Determining requirements for patient-centred care: a participatory concept mapping study. BMC Health Serv Res.

[27] Blain-Moraes S, Racine E, Mashour GA. Consciousness and Personhood in Medical Care. Front Hum Neurosci. 2018;12. 10.3389/fnhum.2018.00306. Frontiers. 10.3389/fnhum.2018.00306 PMC6082939 30116185

[28] Klein C, and Jakob Hohwy. Variability, convergence, and dimensions of consciousness. In: Overgaard M, editor. Behavioral methods in consciousness research. Oxford University Press; 2015. pp. 249–64. 10.1093/acprof:oso/9780199688890.003.0014.

[29] Kaufman SR (2000). In the Shadow of “Death with Dignity”: Medicine and Cultural Quandaries of the vegetative state. Am Anthropol.

[30] Kaufman SR (2003). Hidden places, uncommon persons. Vulnerable Places: Contextualizing Health Practices.

[31] Donis J, Bernd, Kräftner (2011). The prevalence of patients in a vegetative state and minimally conscious state in nursing homes in Austria. Brain Injury.

[32] Catley P, Pywell S, Tanner A (2021). End-of-life decisions for patients with prolonged Disorders of consciousness in England and Wales: Time for Neuroscience-informed improvements. Camb Q Healthc Ethics.

