# Supplementary material for: Perspectives on consciousness in patients with disorders of consciousness from brain injury: group concept mapping study across clinic, research, and families
Source: BMC Health Serv Res. 2023 May 10;23:471. doi: 10.1186/s12913-023-09438-z (PMC10173477; doi:10.1186/s12913-023-09438-z)
Supplement: Supplementary file 1 — Supplementary Material 1 [file 12913_2023_9438_MOESM1_ESM.pdf]

Supplementary Material

Appendix A. Cluster and statement report. GCM study of perspective on consciousness, Denmark 2022

Cluster	Ideas #		Ratings of importance (median)
1 Presence			
n=11	2	That the patient perhaps rather briefly shows signs of a wakeful state with eye contact. **(Moved from Cluster 2)**	3
	3	That the patient perhaps rather briefly shows signs of a wakeful state with recognition.	4
	21	An ability to receive information from one's environment.	3.5
	22	An ability to receive stimuli from one's environment.	3
	32	Spontaneous physical activity.	2
	41	A condition that varies over time.	3
	42	To sense the passage of time.	3
	44	To sense one's environment.	4
	54	Open eyes with depth.	3
	62	Wakeful state.	2.5
	77	A sense of eye contact. **(Moved from Cluster 2)**	3.5
2 Intentional activity			
n=9	4	That the patient perhaps rather briefly shows signs of a wakeful state with intentional activity.	4
	10	When you are certain that the reaction the patient exhibits is not a reflexive reaction, but an intentional movement. E.g. being able to achieve eye contact.	4
	11	When you are certain that the reaction the patient exhibits is not a reflexive reaction, but an intentional movement. E.g. being able to make some trailing motions.	3
	18	That the patient's response appears to be intentional.	4
	51	Selective movements, that can be differentiated from reflexes.	3.5
	52	Trailing motions with the eyes.	3.5
	53	Eye fixation.	2,5
	57	Some comparatively certain (repeated) reaction, such as eye contact.	4
	65	Reliable eye contact **(Moved from Cluster 1).**	3.5
3 Experience of self			
n=12	7	That one is aware of one's own thinking.	3
	9	Being capable of thought processing, which process sensory stimuli from the physical environment, providing a feeling of existence.	4
	23	A capability to process information.	3.5
	24	A capability to process stimuli.	3
	34	A capability to relate to one's own sensory impressions.	4
	35	A capability to relate to one's own thoughts.	4
	39	A condition that is necessary to be able to engage in meaningful activities.	4
	40	A condition that is necessary to participate in one's own life.	4
	45	To be conscious of one's own consciousness.	4
	46	To be reflective of one's own consciousness.	3
	61	Cognition.	4
	63	The capability to be. **(Moved from Cluster 1)**	3
4 Participation in social interaction			
n= 13	1	Participation. **(Moved from Cluster 1)**	3.5
	25	An ability to indicate that information has been processed.	4
	26	An ability to indicate that stimuli has been processed.	3.5

	27	An ability to react to some form of stimuli in a way that is not reflexive, but indicates thought activity.	4
	43	Being able to interact with one's surroundings.	4
	47	Any form of intentional attempt to communicate, both verbally and non-verbally (also if it is not functional).	4
	48	Any reproducible sign of consciousness towards the self.	3
	59	Relevant interaction.	4
	81	The ability to interact socially by mobilizing knowledge- and experience capabilities based on sensorial inputs.	4
	82	The ability to interact socially by mobilizing knowledge.	4
	83	The ability to interact socially by mobilizing experience.	4
	84	The ability to interact socially by mobilizing abilities based on sensorial inputs.	3
	85	The integration of emotional, motor and socially adjusted response for mutual benefit and enjoyment.	3.5
5 (Repeated) response			
n=19	5	That there is a response to some extent. It may be on multiple levels.	3
	6	That there is a response to some extent, and preferably reproducible.	3.5
	8	That one systematically reacts to different sensory stimuli, which cannot be ascribed to reflexive patterns. **(Moved from Cluster 6)**	4
	12	When you are certain that the reaction of the patient is not reflexive, but an intended movement. E.g. communication through blinking.	4
	13	When you are certain that the reaction of the patient shows, is not reflexive, but an intended movement. E.g. communication by use of the thumb.	4
	14	When you are certain that the reaction of the patient is not reflexive, but an intentional movement. E.g. a small movement of the leg upon request.	4
	16	That the patient responds to the stimuli that is provided.	3
	17	That the patient's response is reproducible.	3.5
	19	That the patient's response is consequential, even though it is not related to the type of stimuli that is given.	3
	20	A non-random reaction to external stimuli that can be registered by an examiner.	3.5
	28	The ability to react to some kind of stimulus in a way that is not reflexive but reflects a form of mirroring.	3
	29	The ability to react to some kind of stimulus in way that is not reflexive but reflects a form of interaction.	4
	37	That the person reacts adequately to external sensory stimuli.	4
	49	Any reproducible sign of awareness towards the environment inhabited by the individual. **(Moved from Cluster 2)**	4
	50	Reproducible reaction on request.	4
	55	Some form of relatively certain (repeated) reaction, such as eye turning.	3
	60	Response.	4
	64	The ability to follow requests. **(Moved from Cluster 4)**	4
	75	Reaction to a verbal request.	4
6 Unspecific reaction			
n=21	15	That the patient reacts to the given stimuli.	3
	30	Tonus reduction by external stimuli, such as touch.	3
	31	Tonus search by external stimuli, such as touch.	3
	33	Reaction not reflex to external stimuli.	4
	36	Reaction to external stimuli. **(Moved from Cluster 5)**	3
	38	That the person reacts to external stimuli. **(Moved from Cluster 5)**	3
	56	Some form of relatively certain (repeated) reaction, like changes in breathing when touched.	3
	58	Some form of relatively certain (repeated) reaction, like increased pulse when stimulated.	3

	66	Reaction to stimuli by movement.	3
	67	Reaction to stimuli by mimicking.	3
	68	Reaction to stimuli by eye reactions that are registered on monitoring equipment.	3
	69	Reaction to pain stimuli, that as a minimum averts, but it is not reflexive.	3
	70	Reaction on the Glasgow Coma Scale.	3
	71	Reaction to sensory stimuli, e.g. sounds.	3
	72	Reaction to sensory stimuli, e.g. touch.	3
	73	Reaction to sensory stimuli, which can be registered in the pulse.	2,5
	74	Reaction on sensory stimuli, which can be registered on vasomotor tonus.	3
	76	Tears.	1
	78	Reaction to verbal contact. The reaction may be in the form of small movements (eyelids, twitches by the mouth, fingers/toes that move.) These reactions should increase in a matter of days / weeks.	3
	79	Reaction to touch. The reaction may be in the form of small movements (eyelids, twitches by the mouth, fingers/toes that move.) These reactions should increase in a matter of days / weeks.	3
	80	Reaction to being moved to the outdoors. The reaction may be in the form of small movements (eyelids, twitches by the mouth, fingers/toes that move.) These reactions should increase in a matter of days / weeks.	3
